# Treatment of endoscopic ultrasound‐guided coil deployment for isolated gastric varices using 0.035‐inch hydrocoil: Experience of three cases

**DOI:** 10.1002/deo2.252

**Published:** 2023-06-15

**Authors:** Kazunori Nagashima, Ken Kashima, Yasuhito Kunogi, Fumi Sakuma, Koh Fukushi, Akira Yamamiya, Yoko Abe, Keiichi Tominaga, Makoto Iijima, Kenichi Goda, Rafael Romero-Castro, Atsushi Irisawa

**Affiliations:** ^1^ Department of Gastroenterology Dokkyo Medical University School of Medicine Tochigi Japan; ^2^ Endoscopy Unit Gastroenterology Division Virgen Macarena University Hospital Seville Spain

**Keywords:** EUS‐coiling, EUS‐guided coil deployment, hydrocoil, interventional ultrasound, isolated gastric varices

## Abstract

Endoscopic ultrasound (EUS)‐guided coil deployment (EUS‐coiling) has been newly developed for treating isolated gastric varices (iGV). This report describes three cases of EUS‐coiling for iGV using a 0.035‐inch hydrocoil (Azur; Terumo Corp., Tokyo, Japan). When used for EUS‐coiling, this hydrocoil provides the following benefits: Its electrically detachable system allows pull back. It has smooth and dense deployment. Moreover, it has a strong blood‐flow blocking effect because of its long length and large diameter with internal swelling functions of the hydrogel. Technical success of coiling was achieved in all cases. After coiling, additional treatments such as cyanoacrylate and sclerosant injection were performed as deemed appropriate. All iGVs were obliterated successfully. No adverse event occurred during the procedure or during the mean follow‐up of six months. Our findings indicate that this 0.035‐inch hydrocoil can be used to treat iGV safely and effectively.

## INTRODUCTION

Recently, interventional endoscopic ultrasound (EUS) has made remarkable progress. Along with that development, EUS‐guided coil deployment (EUS‐coiling) is now being performed for isolated gastric varices (iGV).^1^ Additionally, we perform EUS‐guided coil deployment with sclerotherapy, for which ethanolamine oleate is injected as a sclerosant after EUS‐coiling, based on the presumption that achieving eradication is important not only for iGV but also for feeders to prevent a recurrence.^2^ Difficulties related to coils (e.g., wool coils) that have been used in the past are that they cannot be pulled back, the coil might kink in the needle, and many coils must be used for sufficient blood flow control. Recently, hydrocoils (Azur; Terumo Corp., Tokyo, Japan) have become available for vascular embolization. One report has described the use of a 0.018‐inch hydrocoil in EUS‐coiling for iGV.^3^ However, using EUS‐coiling with a large‐diameter 0.035‐inch hydrocoil has not been described. This report describes three cases of EUS‐coiling applied for iGV using a 0.035‐inch hydrocoil.

## METHOD

The EUS‐coiling procedure has been approved at Dokkyo Medical University Hospital (Approval number 2019‐012) for examination with permission to provide difficult new medical technologies. This study, conducted in accordance with the ethical principles associated with the Declaration of Helsinki, was approved by the institutional review board of Dokkyo Medical University Hospital (Approval number R‐45‐3J). Our website provides patients a means to opt out of research participation instead of omitting informed consent, which guarantees research subjects an opportunity to avoid the reporting and publishing of their related research information. The evaluation outcomes were used to assess the feasibility and safety of the hydrocoil deployment.

**FIGURE 1:**
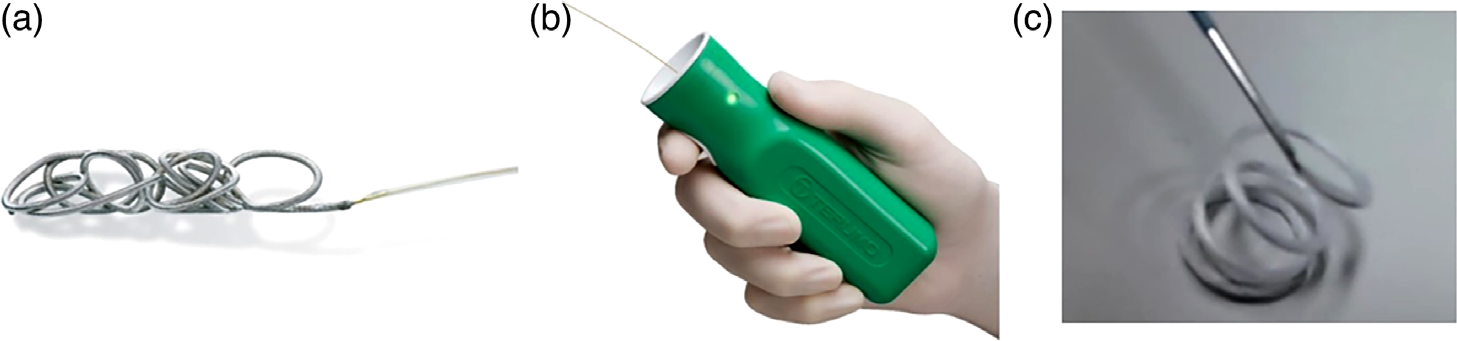
(a) Hydrocoil (Azur; Terumo Medical Corp. Tokyo, Japan). (b) The coil and sheath were detached using this electrically detachable system. After the sheath end is inserted into the green handle, a current is applied for disconnection. (c) Wool coil (MReye; Cook Medical, Tokyo, Japan).

**FIGURE 2:**
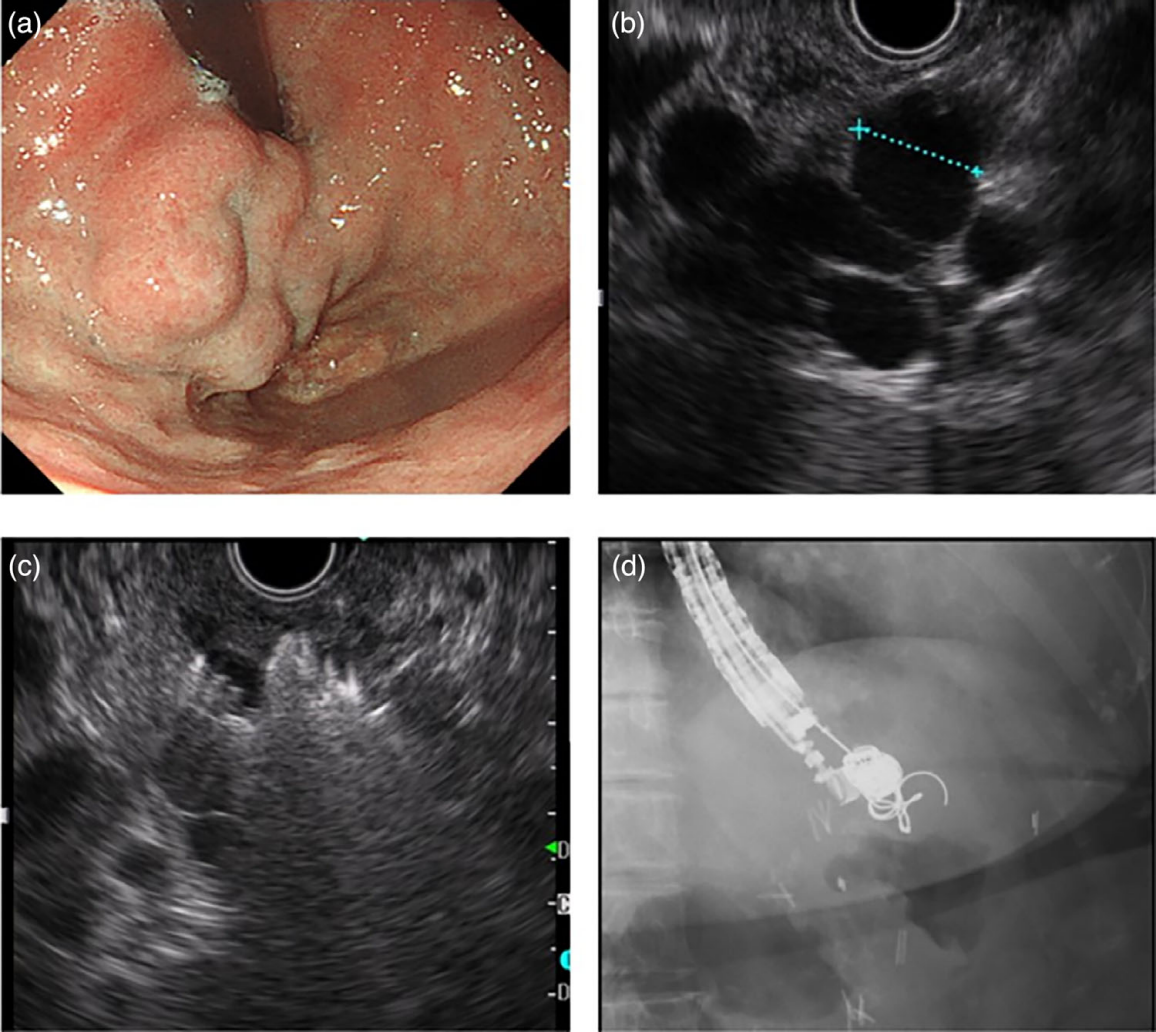
(a) Endoscopy showed Lg(f). (b) Endoscopic ultrasound (EUS) showed a varix diameter of about 12 mm. (c) Coil placement can be confirmed using EUS. (d) Fluoroscopic image after treatment. Fluoroscopy results confirmed that the coil is placed tightly.

EUS‐coiling was performed using a convex‐arrayed EUS (GF‐UCT 260; Olympus Corp., Tokyo, Japan). We visualized the splenic vein from within the stomach and checked for iGV that are continuous from the feeders. After using EUS to confirm the target iGV from the abdominal esophagus, the maximum diameter of the iGV to be punctured was measured. The iGV was punctured with a 19‐gauge EUS‐guided fine‐needle aspiration needle (Expect; Boston Scientific Japan, Tokyo or EZ shot3 plus; Olympus Corp.). Subsequently, a 0.035‐inch hydrocoil (Figure 1a) with a diameter that was 150%–200% of the iGV diameter was inserted into the needle lumen. After coil deployment with complex entanglement in the iGV, the coil and sheath were detached using an electrically detachable system (Figure 1b) and were deployed completely in the iGV by pushing the sheath. An additional hydrocoil or wool coil (MReye; Cook Medical, Tokyo, Japan; Figure 2c) was placed if color Doppler imaging showed residual blood flow. The procedure described above was repeated until no signal of blood flow was found by color Doppler imaging. Finally, sclerosant (ethanolamine oleate) was injected into the iGV until an image of feeders under fluoroscope guidance was obtained. In addition, a tissue adhesive substance (cyanoacrylate) was injected if necessary.

Technical success was defined as the release of the hydrocoil completely into the iGV. Clinical success was defined as the disappearance of iGV.

## CASE REPORT

This case series includes three cases of EUS‐coiling for iGV. A summary of the three cases is presented in Table 1.

**TABLE 1:** Summary of three patients who underwent endoscopic ultrasound‐guided coil deployment for isolated gastric varices using 0.035‐inch hydrocoil.

Case	Age	Sex	Underlying disease (Medical history)	Child‐Pugh (Class/Score)	Varices size(mm)	Needle	First coil (mm × cm)	No. of coils in use	Amount of 5%EO (ml)	Success (Technical/Clinical)	AdditionaCA/EO injection	Adverse events	Follow‐up period (month)	Outcome
1	75	F	Cirrhosis (AIH)	A/5	12	19G EZ‐shot3	20 × 39	4	5	Yes/Yes	None	None	8	No recurrence Alive
2	72	F	Cirrhosis (NASH) Diabetes	A/6	10	19G Expect	16 × 19	3	3	Yes/Yes	Yes	None	6	No recurrence alive
3	83	F	Cirrhosis (HCV) Hypertension	A/5	11	19G Expect	16 × 19	3	0	Yes/Yes	Yes	None	4	No recurrence alive

Abbreviations: AIH, autoimmune hepatitis; CA, cyanoacrylate; EO, ethanolamine oleate; HCV, hepatitis C virus; NASH, nonalcoholic steatohepatitis.

### Case 1

A 75‐year‐old woman had a 12‐mm‐diameter iGV (Figure 2a,b). Four hydrocoils (20 mm × 39 cm, 16 mm × 32 cm, 13 mm × 24 cm, and 8 mm × 24 cm) were deployed (Figure 2c,d). Then, 5 ml of 5% ethanolamine oleate was injected immediately to obliterate the remaining small varices and feeders.

### Case 2

A 72‐year‐old woman had a 10‐mm‐diameter iGV. The first coil was a 16 × 32 cm hydrocoil, followed by two additional 10 mm × 10 cm wool coils. Subsequently, 3 ml of 5% ethanolamine oleate and 1.4 ml of cyanoacrylate were injected to obliterate the remaining small lumen and feeders.

### Case 3

An 83‐year‐old woman had an 11‐mm‐diameter iGV. Three hydrocoils (16 mm × 32 cm, 16 mm × 32 cm, and 10 mm × 19 cm) were deployed. Subsequently, 1.4 ml of cyanoacrylate was injected to obliterate the remaining small lumen and feeders.

All cases had technical and clinical success. No adverse event was observed in any case during the procedure or during the 6‐month follow‐up.

## DISCUSSION

For EUS‐coiling for iGV, a 0.035‐inch hydrocoil is expected to be adequately safe, based on our preliminary experience. A 0.035‐inch wool coil has been used as the standard coil for the EUS‐coiling of iGV. However, pulling back a wool coil is impossible. Moreover, it might kink in the needle during the procedure. In addition, during the treatment of EUS‐guided coil deployment for iGVs using a wool coil, leakage of the coil into the systematic circulation has been reported.^4^, ^5^ Based on these considerations, a deployed first coil is expected to have a shape that reliably occupies the inner area of iGV with a winding shape.

**FIGURE 3:**
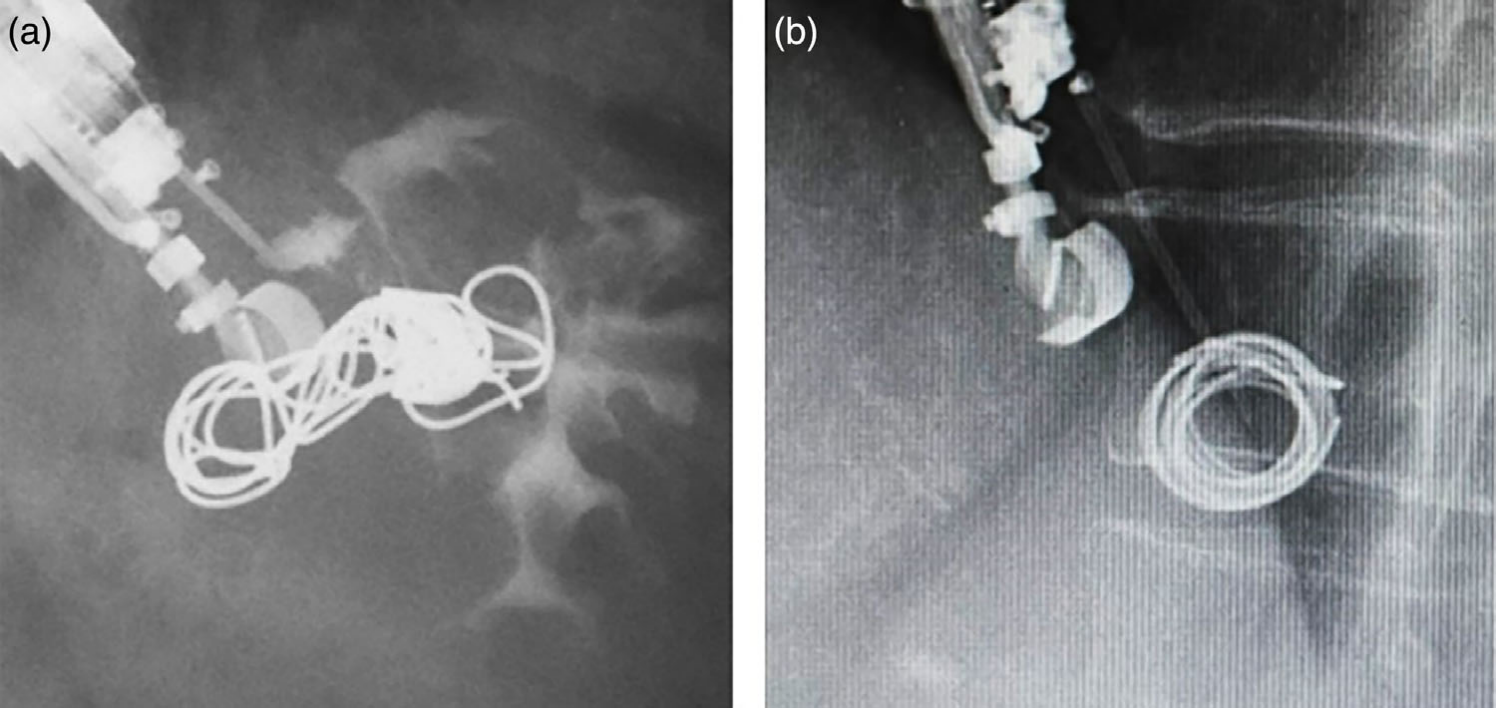
The hydrocoil in panel (a) is shown for comparison to the wool coil in panel (b). Hydrocoils are deployed more densely.

The 0.035‐inch hydrocoil used this time employs an electrically detachable system that allows pulling back between the coil and the delivery sheath. Because this hydrocoil has no synthetic fibers, it does not interfere easily with the needle tip. For these reasons, it is possible to achieve smooth deployment with no kinking. Moreover, this hydrocoil is characterized by its long coil length and thick diameter. It is dense, with a strong radially expanding placement form compared to the characteristics of the wool coil (Figure 3a,b). Because it has a 0.035‐inch thickness and the internal swelling function of the hydrogel, few gaps form between the coils. Therefore, theoretically, the 0.035‐inch hydrocoil is regarded as having a stronger radial expansion force and blood‐flow blocking effect than either the 0.018‐inch hydrocoil or the wool coil. Mosquera‐Klinger et al. reported EUS‐coiling for iGV using a 0.018‐inch hydrocoil.^3^ As found from the case series described herein, although no adverse event was reported, variceal blood‐flow blocking, dense placement, and the radial expansion force effect of a thick 0.035‐inch coil might be stronger than those of a 0.018‐inch coil. Based on these findings, we consider that the 0.035‐inch hydrocoil standard prevents migration. Earlier, we reported EUS‐coiling using a conventional coil.^2^ As reported herein, the average number of coils used in eight cases was 5.6. In contrast, the average number of 0.035‐inch hydrocoils used was 3.3. These findings suggest that the 0.035‐inch hydrocoil is highly effective at blocking blood flow and that it might lead to a reduction in the number of coils.

The 0.035‐inch hydrocoil benefits include its 1) smooth deployment without kinking because of the pullback function and 2) strong radial expansion force and dense deployment attributable to its large diameter and coil length. Its internal swelling function can be expected to reduce migration and the number of coils. We regard its only shortcoming as its high cost ($1030), which is 10 times that of MReye ($103). In light of the benefits described above, we think that it is more than worth the price.

In conclusion, a 0.035‐inch hydrocoil presents numerous beneficial features. It is anticipated for use with EUS coiling because of its safety and effectiveness.

## CONFLICT OF INTEREST STATEMENT

Rafael Romero‐Castro has been a consultant to Cook Medical since February 28, 2023.

## ETHICS STATEMENT

This case series report was conducted in accordance with the ethical standards established in the 1964 Declaration of Helsinki and its later amendments.
